# Adolescents’ Suggestions on how to Support Their Sleep: A Focus Group Study

**DOI:** 10.1177/10598405221084317

**Published:** 2022-03-07

**Authors:** Malin Jakobsson, Karin Josefsson, Karin Högberg

**Affiliations:** 1 Faculty of Caring Science, Work Life and Social Welfare, 1802 University of Borås, Sweden; 2 Department of Health Science, 4209 Karlstad University, Sweden

**Keywords:** sleep, sleeping difficulties, adolescent, support, health-promotion, school nurse, parents, focus group, qualitative content analysis

## Abstract

Sleeping difficulties among adolescents constitute a pressing public health issue, and it is of the utmost importance that these are approached from a health-promotion perspective. This study explores adolescents’ suggestions on how their sleep could be supported. Data were collected via eight focus group interviews with 43 adolescents aged 15–16, in Sweden, and analyzed using qualitative content analysis. The results describe the adolescents’ suggestions on how their sleep could be supported in three themes: being supported by involved parents— routines, engagement, and warmth are vital; being supported to achieve knowledge on the “whys” and “hows” of sleep—increased knowledge gives the ability to make well-grounded sleep choices; and being guided into finding balance—balance in life is difficult to achieve and adolescents desire support. Parents, school nurses, health professionals, and anyone who has the opportunity to improve and promote sleep should consider adolescents’ suggestions.

## 1. Introduction

Now recognized as a public health issue, sleeping difficulties among adolescents, mainly involving insufficient sleep, difficulties initiating sleep, daytime sleepiness, and poor sleep quality are common (Gradisar et al., 2011; Louzada, 2019; Saxvig et al., 2021). A study with 165,739 adolescents throughout Europe and North America showed that, while the proportion varies between countries, only between 32% and 86% of adolescents get their recommended eight hours of sleep (Gariepy et al., 2020). The consequences of sleeping difficulties include negative school performance, increased risk-taking behaviors, psychosocial health problems, somatic health problems (Shochat et al., 2014; Sun et al., 2019), and negative emotional reactivity (Tempesta et al., 2020). To avoid persistent sleeping difficulties, health-related problems, and school failures, it is of the utmost importance to promote good sleep among adolescents. A Norwegian study has emphasized the need for early identification of this problem, as 68% of adolescents with insufficient sleep between the ages 16–18 had maintained the same unhealthy sleep patterns when surveyed six years later (Hysing et al., 2020).

Adolescents’ sleeping difficulties may be affected by their adolescence, with its biological, developmental, and socio-environmental changes (Carskadon, 2011). Biological factors, such as a circadian shift and slowing of sleep homeostatic pressure, naturally delay sleep onset and waking time in puberty, making it difficult for adolescents to get enough hours of sleep before school (Carskadon, 2011; Crowley et al., 2018). During adolescence, academic and social demands increase, while, at the same time, parental supervision decreases (Becker et al., 2015; Crowley et al., 2018). Adolescents’ health conditions such as anxiety, depression, and pain may interfere with their sleep (Orchard, 2020; Shochat et al., 2014), and external circumstances, such as social norms, values, and requirements from family, friend groups, school, social media, and society can make sleep challenging (Jakobsson et al., 2022).

Other factors that are seen to be related to sleeping difficulties include pre-sleep worrying, evening light, the use of technology other than television, the use of tobacco, caffeine, and alcohol, a negative family environment (Bartel et al., 2015), and school stress (Gaarde et al., 2020; Jakobsson et al., 2019, Jakobsson et al., 2020). Factors known to promote sleep among adolescents include physical activity, daylight, routines, regularity, screen regulation before bedtime, parents’ support, and a good family environment (Bartel et al., 2015; Khor et al., 2021; Machado et al., 2020).

There is evidence that the adults closest to adolescents are most important for supporting their sleep (Khor et al., 2021; Machado et al., 2020). The question is how adults can support adolescents’ sleep before they are in need of professional care and treatment. Adult educators and school nurses may also promote adolescents’ sleep, since they interact naturally with them in school. Numerous school interventions have been tested, usually aimed at increasing adolescents’ knowledge about sleep and/or generating positive changes in their sleep behavior, such as sleep hygiene, bedtimes, and regularity. The design and length of the interventions vary, from a single session to recurring sessions, with different forms of education, such as sleep knowledge, motivational factors, sleep diaries, and parental involvement. The strategies for changed sleep behavior have been based on, for instance, motivational interviews, cognitive behavioral theory, time and stress management, and bright light therapy. The approaches vary, including slides, leaflets, workshops, workbooks, interactive groups, and seminars (Blunden & Rigney, 2015; Gruber, 2017; Illingworth, 2020). School-based sleep education in classes is effective in increasing adolescents’ knowledge about sleep; however, it has been less effective in improving sleep behavior (Blunden & Rigney, 2015; Gruber, 2017; Illingworth, 2020). For school interventions to effectively modify sleep behaviors, great consideration must be given to pedagogical approaches, quality assurance (Bunden & Rigney, 2015), and the adolescents’ own motivations (Illingworth, 2020).

There is a need for studies with different methodological perspectives to explain and understand adolescents’ sleep (Becker, Langberg & Byars, 2015). However, there is a lack of qualitative research in which the adolescents’ own experiences are explored. Adolescents’ own experiences are important for future interventions and health promotions to be more successful in preventing sleeping difficulties. All too often, adults believe they know adolescents’ needs without asking them (Coyne et al., 2016). Thus, it is important to explore adolescents’ suggestions on how their sleep could be supported, which is this study’s aim. This study can contribute to forming the basis for how the school system, school nurses, and health professionals can promote adolescents’ sleep.

## 2. Method

### 2.1. Design

To address the aim of this study, an inductive qualitative design was adopted. A qualitative content analysis developed by Lindgren et al. (2020), which strives to make the participants’ voices heard, was used. This analysis utilizes an interpretative approach inspired by the hermeneutic paradigm (Lindgren et al., 2020). When data allow for the interpretation of latent content, such as in the current study, qualitative content analysis can reveal both the depth and meaning of participants’ narratives. Focus group interviews were chosen to explore adolescents’ suggestions on how their sleep could be supported. Focus group interviews are especially useful for gaining insight into a target group’s experiences and perceptions regarding an issue, an idea, or a phenomenon. According to Krueger and Casey (2015), the ideal size of a focus group is between five and eight participants; a focus group offers a valuable and versatile format that gives the participants an opportunity to interact and reflect. The consolidated criteria for reporting qualitative research (COREQ) were followed (Tong et al., 2007). The Swedish Ethical Review Board approved this study (Dnr: 2019-03806).

### 2.2. Participants

The participants were comprised of *n* = 43 adolescents divided into eight focus groups, with 18 boys (42%) and 25 girls (58%). The participants were in grade nine, aged 15–16, from five socioeconomically diverse schools in a Swedish city (Table 1). The schools are located in rural, high-status, and vulnerable areas. Grade nine in Sweden is the last year of secondary school prior to three years of upper secondary school. The school start time in Sweden is usually between 8:00–8:30. There were no inclusion and exclusion criteria. Accordingly, both those with and without sleeping difficulties were included, since all humans have their own experiences of needs and support for their sleep. Nevertheless, all participants described current or past experiences of insufficient sleep and difficulties falling asleep. 

**Table 1.** Overview of Focus Groups (n = 8) and Participants (n = 43).

Focus groups	Participants	Live with cohabiting parents	Have siblings	Foreign origin	Do leisure exercise	Have friends in leisure
	*n* = 43	*n* = 35 (81%)	*n* = 42 (98%)	*n* = 16 (37%)	*n* = 31 (72%)	*n* = 42 (98%)
1	4 boys	4	4	2	3	4
2	1 boy, 5 girls	3	6	5	4	6
3	5 boys, 1 girl	5	5	1	4	6
4	5 girls	4	5	2	3	5
5	5 girls	5	5	4	2	4
6	6 girls	5	6	0	6	6
7	2 boys, 2 girls	4	4	0	4	4
8	6 boys, 1 girl	5	7	2	5	7

### 2.3. Procedure and Data Collection

To collect data, heads of school administrations, principals, and teachers were contacted and informed about the study orally and in written form. Only the schools that gave permission to conduct the study were included. One class per school was visited to inform the adolescents about the study orally and in written form. The teachers in grade nine at each school agreed on which class was suitable to participate based on scheduling opportunities. A list was left in each classroom on which interested adolescents wrote their names. In cases where the number of registered adolescents could be divided into two focus groups, the teacher, who was familiar with which peer groups they felt safe in, performed that action. All focus groups (*n* = 8) were conducted during school days from December of 2019 to January of 2020. Because the adolescents were in the same class, their familiarity created an open environment in which conversation flowed easily. The first author led the conversations and the last author participated as an observer and reflected points that the first author might have forgotten to mention.

The interviews were guided by three semi-structured questions: “Can you please tell me what you need for your sleep?”; “Can you please tell me what support you need for your sleep?”; and “Can you please tell me how you want the support to be designed?” The adolescents were encouraged to narrate their experiences and suggestions as freely as possible. The open-ended follow-up questions, “How?”; “When?”; and “Can you give an example?” were used to clarify and encourage further narration. The audio-recorded interviews lasted 32–50 min and were transcribed verbatim.

### 2.4. Data Analysis

Data were analyzed using qualitative content analysis with an inductive approach; the themes were not determined in advance but gradually emerged during the analysis (Graneheim & Lundman, 2004; Lindgren et al., 2020). Depending on the study’s aim and the quality of the data, this method enables manifest content to be identified, then goes on to abstracts and interprets the latent content of the underlying meanings that run through the text, and finally formulates sub-themes and themes (Lindgren et al., 2020).

In the first step of the analysis process, the data in the form of the original transcript were read repeatedly in their entirety. In the second step, the data were de-contextualized into meaning units, meaning that words and sentences pertaining to the aim of the study were marked and lifted out of the text. In the third step, the meaning units were condensed and coded, while the original transcript was read in parallel to ensure that its core was still preserved, which is an aspect of credibility. Thereafter, during re-contextualization, the codes were carefully sorted into groups based on similarities and differences. Here, the researcher went beyond the exact words of the text and was open to the emotions and underlying meanings conveyed. During this stage of the analysis process, the researcher moved back and forth between the whole original text and its parts to ensure trustworthiness in the interpretations (Graneheim & Lundman, 2004). Threads of meaning gradually emerged from the group of codes and formed nine sub-themes that were further interpreted and abstracted into three themes. Table 2 provides an example of the data analysis. To ensure trustworthiness, MJ and KH discussed similarities and differences and agreed on how to code the same content. The codes, sub-themes, and themes were thoroughly discussed with KJ and a consensus was reached in the research group. Finally, the results were discussed with research colleagues in seminars. 

**Table 2.** An Example of the Qualitative Content Analysis.

Meaning unit	Condensed meaning unit	Code	Sub-theme	Theme
I think that if from childhood you get well-structured sleep; that is absolutely what is most important, as when you enter adolescence, it becomes harder and harder for most people, I think	from childhood get well-structured sleep, is most important, as when you enter adolescence, it becomes harder	Structuring sleep from childhood	Having family routines	Being supported by involved parents

### 2.5. Ethical Considerations

Ethical research principles were carefully followed by fulfilling the requirements of information, consent, confidentiality, and usage (World Medical Association, 2013). In accordance with the Swedish Code of Statutes (2003:460, § 18), if a participant is between 15 and 18 years of age and has the ability to independently understand what the research entails, he or she can consent to be included without parental consent. All adolescents were aged 15–16 and gave written informed consent to participate in the study.

## 3. Results

The results describe the adolescents’ suggestions of how their sleep could be supported in three themes: being supported by involved parents, being supported to achieve knowledge on the “whys” and “hows” of sleep, and being guided into finding balance, each of these with three sub-themes. Table 3.

**Table 3.** Overview of Themes, sub-Themes, and Quotes of Adolescents’ Suggestions on how Their Sleep Could be Supported.

Themes	Sub-themes	Quotes
Being supported by involved parents	Having family routines	*“…getting a good structure on sleep from childhood is absolutely most important. When you enter adolescence it becomes more and more difficult”* (Focus group 1) *“Although it's hard when the parents come and say: ‘Okay, now it's quite late, we are going to bed now, do not stay up too long… that they still are coming, that they still care is important…”* (Focus group 4)
	Having a sense of security	*“…I need a little sound before I fall asleep… I cannot fall asleep otherwise, when Mom and Dad sit and watch TV, I calm down because I hear that someone else is awake…”* (Focus group 3) *“…it is not always good at school and you may think about it in the evenings, but if you have talked to your parents about it then maybe it can be a little easier…”* (Focus group 7)
	Having a sleep-friendly nest	*“…to have somewhere to sleep, and that it should be quiet…”* (Focus group 5) *“…Sleep alone, and have clean sheets…”* (Focus group 2)
Being supported to achieve knowledge on the “whys” and “hows” of sleep	Being motivated to gain personal insight	*“…I have always heard from Mom and Dad that it is important to sleep but we have never really gone into why or what the consequences can be… even if I can figure out a little myself…”* (Focus group 7) *“…I have compared being up as long as I usually am (1 AM) and felt how tired I am in the morning, then I have gone to bed at eleven and am still just as tired. It does not feel like it helps to fall asleep early… so it doesn't matter… I get alert in the evening at ten or eleven…”* (Focus group 3)
	Acquiring knowledge from those you trust	*“…I do not care if an "unknown" person says something but more what my mother says…”* (Focus group 5) *“…if my parents learned from the school nurse and then can give me better tips…”* (Focus group 3)
	Being interactive during sleep education	*“…to hold a discussion about what you think happens if you do not sleep and so you can learn by discussing…”* (Focus group 8) *“…you do not listen as much to a person who you have just gotten to know. So it is important for the school nurse or the teacher to first get to know the students…”* (Focus group 5)
Being guided into finding balance	Being encouraged to participate in activities	*“…if you have exercised after school, like the gym and then showered…* *then you can sleep easily. But if you are only at your mobile after school then it is difficult to sleep…”* (Focus group 2) *… I do not have much to do after school… then I usually sleep after school and do not fall asleep in the evening… but it is just a habit and I cannot control it* *…* *maybe it would be easier if we came up with some activity in the family…”* (Focus group 5)
	Learning to deal with silence	*“…I usually try to turn off the light and lie still and think of nothing… but it does not work so I pick up the phone until I get tired…”* (Focus group 8) *“…I usually put on a podcast before I go to sleep because I cannot be in completely silence, I panic, I do not know how to do otherwise… I need help to find a solution…”* (Focus group 4)
	Being supported with a structure in school	*“…I know you have to take your own responsibility, but it can be difficult* *…* *so maybe the teachers can push on and help you a little with the structure and not just say which day you should be ready…”* (Focus group 7) *“…the school can help by giving fewer tests, assignments, and homework, or at least not all in the same week…”* (Focus group 2)

### 3.1. Being Supported by Involved Parents

The adolescents suggested that parents should be involved, as this contributes to better sleep. Parental involvement can be expressed in different ways: having family routines, having a sense of security, and having a sleep-friendly nest.

#### 3.1.1. Having Family Routines

Adolescents expect parental direction in daily routines. Having routines integrated during childhood is experienced as valuable, since routines are more difficult to establish later in life. Routines that support sleep include parent-implemented practices regarding eating before bedtime, limiting mobile phone use, having set bedtimes, unwinding in the evening, and regularity. Adolescents want to participate more in these decisions as they age. Having a routine may also involve the parents looking into the adolescent’s bedroom before going to sleep and waking them in the morning.

“…even though it’s hard for the parents to remind and nag us to go to bed and put down the phone… you need someone to tell you to do it…” (Focus group 6)

#### 3.1.2. Having a Sense of Security

Adolescents want parents to promote a home and family that offers a sense of security; this encourages sleep. A sense of security makes it easier to relax and fall asleep rather than feeling lonely and anxious. Such peaceful sentiments are connected to hearing that the parents are awake, perhaps watching TV or doing the laundry. A sense of security also occurs if the door from the bedroom to the hall is ajar, a lamp gleams, they are aware that the parents always lock the front door, or they have a cuddly toy to hug. When parents ask how the day has been and listen to their concerns, the adolescents feel secure and supported when it is time to sleep.

“…my father said to me that having a cuddly toy next to you helps most children sleep because then you do not feel alone… and it actually helps… you feel safe… sometimes when I have difficulty sleeping, I sleep with a cuddly toy… even though I’m 16…” (Focus group 4)

#### 3.1.3. Having a Sleep-Friendly Nest

Adolescents suggest parents should be involved in the creation of a sleep-friendly nest to set the conditions for a good night’s rest. A sleep-friendly nest, which gives a feeling of calm, consists of one’s own bed and, preferably, one’s own cozy bedroom. Other supportive factors for experiencing the bedroom as sleep-friendly include peace and quiet there, blinds that allow the room to remain dark, a cool temperature, and bedsheets that are changed regularly.

*“…you need peace and quiet, where it is dark, no sounds, to be relaxed… a calm environment…”* (Focus group 1)

### 3.2. Being Supported to Achieve Knowledge on the “Whys” and “Hows” of Sleep

Adolescents have less information about the “whys” and “hows” of sleep than they would like; they require more knowledge. Knowledge that motivates personal insight is acquired from those you trust and is gained through being interactive during sleep education.

#### 3.2.1. Being Motivated to Gain Personal Insight

For knowledge to play a role in sleep decisions, adolescents wish to be motivated by personal insight. They want to take personal responsibility for their sleep, but recognize that their knowledge about sleep is limited. A superficial understanding of the consequences of too little sleep does not motivate decisions that promote sleep; for instance, it is difficult to choose to put down the mobile phone in favor of sleep. Adolescents want to understand the sudden, seemingly incomprehensible transformations in their sleep patterns that make it impossible to fall asleep at nine o’clock, when it used to be easy. In order to prioritize sleep, the adolescents feel that personal insight and deeper knowledge are needed.

“…from the outside, adults can always influence and pay attention to things and try to help, but in the end, it is always up to me because only I can decide when I go to bed… you probably have to get to a point yourself where you decide that this is actually what you want to do…” (Focus group 6)

#### 3.2.2. Acquiring Knowledge from Those you Trust

To support their sleep, adolescents prefer to acquire knowledge from those they trust. The best experiences occur when people with whom they are in a relationship of trust deliver knowledge and offer advice. Those who are identified as the sources of influence and the best messengers of knowledge are parents, but older siblings, relatives, and other adults, such as sports coaches, can be helpful too. It creates a sense of confidence when the elders who give advice truly want the best for them. Adolescents suggest that parents should receive sleep education from, for example, the school nurse, in order to acquire additional knowledge with which to support their children.

“…I think you also listen to your parents the most and perhaps have the most respect for them or something like that, and they are at home and they see how you are doing…” (Focus group 6)

#### 3.2.3. Being Interactive During Sleep Education

Adolescents know in which ways they learn best; they suggest that being interactive during sleep education can best support their learning process. The sleep education in schools is largely superficial, focusing only on how much sleep is required. A failure to convey the consequences of poor sleep and how one may find it easier to fall asleep are described by the adolescents. Interactive learning, as opposed to teacher-centered learning, offers the opportunity to learn from each other and hear how friends are reasoning. Furthermore, it is advantageous if the adolescents have an established relationship with the school nurse or the one who leads the sleep education. Being interactive during sleep education could also involve talking about sleep in safe, smaller conversation groups with the school nurse.

“…at school, they always say that you should sleep and how much, but they do not teach you how, they just say that you should sleep… maybe you need a little bit of help with sleep…” (Focus group 1)

### 3.3. Being Guided into Finding Balance

Adolescents want to be independent as well as to receive guidance in finding a balance during the day that will support their sleep. The guidance includes being encouraged to participate in activities, learning to deal with silence, and being supported with structure in school.

#### 3.3.1. Being Encouraged to Participate in Activities

Adolescents wish to be encouraged to participate in activities, since all forms of activity, in moderation, are considered positive and enable a balance between activity and rest. The activity does not have to be in the form of exercise, but could involve hanging out with friends, going to the cinema, or doing something with the family. Even if all forms of activities were experienced as supportive, according to the adolescents, the best sleep occurs when the day includes intense physical activity at school, whether during organized sports training or during leisure time.

“…you can do something with the family. The parents can say, ‘Children, we can do this and that’, it does not have to cost anything… just the act of doing something with the family makes it much easier to fall asleep… because then you have done something…” (Focus group 5)

#### 3.3.2. Learning to Deal with Silence

Adolescents seek support in learning to deal with silence before they sleep. The silence that occurs when they go to bed contrasts with their active and busy days, making it impossible to fall asleep. Lying in a quiet bedroom evokes feelings of panic, discomfort, concern, and boredom. To cope with the silence, calm music, white noise, YouTube, television series, and podcasts are used. On the one hand, listening to or watching something to distract oneself, unwind, and deal with the silence can be considered helpful. On the other hand, those actions easily lead to the unwanted use of a mobile phone at bedtime. The experience is that it is necessary to find a balance between the constant flow of information and silence, in one way or another, in order to fall asleep more easily.

“…throughout the day, you do not think you are surrounded by sound but think it is pretty quiet as well. And then you come home and go to bed and it becomes like a shock factor… Oh God, how quiet… You are little unaccustomed to it and then you cannot sleep…” (Focus group 4)

#### 3.3.3. Being Supported with a Structure in School

To find balance in one’s tasks is important for the coming night’s sleep, and adolescents desire the support of educators in striking this balance between schoolwork and leisure. School days are better organized in the absence of several tests or assignments in the same week. A well-planned schedule of homework, assignments, and tests will reduce adolescents’ stress. In addition, their sleep can be supported by the school if it takes into account that adolescents are generally more tired in the morning, given that the body changes biologically. Their experience is that the whole school day benefits from having sports or other practical lessons first thing in the morning, or having a later starting time.

“…you get more energy if you have sports or practical lessons in the morning than if you just watch movies—then you will be lazy all day…” (Focus group 2)

## 4. Discussion

This study explored adolescents’ suggestions on how their sleep could be supported. The support adolescents see as most helpful are parental involvement, knowledge of the “whys” and “hows” of sleep, and guidance on finding balance. Based on these suggestions, it is likely that schools, school nurses, and other health professionals can provide support that encourages adolescents toward healthy sleep patterns. However, it is not possible to draw the general conclusion that all adolescents want this particular support. On the contrary, the study sheds light on the fact that adolescents have many suggestions for promoting and supporting their own sleep; this should indicate that adolescents’ narratives need to be given attention in caring and in continuing research.

Previous research has shown that support from parents via regular bedtimes, daily routines, and boundaries helps adolescents to sleep (Khor et al., 2021; Machado et al., 2020). In this study, the adolescents themselves emphasized the importance of their parents. Even though the adolescents were aged 15–16 and considered themselves as relatively independent, they constantly returned to the importance of routines, engagement, and warmth from their parents as vital supports. Parental support seemed to bring an invaluable sense of security to the adolescents, making it easier for them to relax and sleep.

The adolescents in this study stated that it is important to have routines from an early age. This is in line with Baker et al. (2019), who described how parents’ ability to guide their children in developing healthy habits is a key to supporting their health in the present and in the long term. In addition to having good health habits from childhood, adolescents can develop the ability to make their own healthy choices during adolescence. According to Moilanen et al. (2018), adolescents’ experiences of autonomy are vital if they are to take responsibility for making healthy choices. This indicates that any health professional working with family care or care directed to adolescents with a preventive purpose should specifically promote healthy habits within the family as a whole, while also strengthening the growing adolescents’ autonomy. Bird et al. (2021) confirmed the advantage of including the family (home) in promotion work. This is because home and school are critical learning environments for successfully shaping students’ sleep behavior. According to the study, a connection between home and school is important when designing future school-based inventions to promote sleep. In sum, the adolescents who have spoken in the present study reinforce previous research on the importance of the family as a source of good sleep habits.

The results highlight adolescents’ requests for knowledge on the “whys” and “hows” of sleep. This increased knowledge could, according to the adolescents, provide personal insights and motivation, thereby giving them the ability to make well-grounded sleep choices. Existing school education interventions demonstrate good opportunities to increase knowledge about sleep (Blunden & Rigney, 2015; Gruber, 2017; Illingworth, 2020). Nevertheless, the adolescents in this study prefer personal and targeted advice and tips over general ones. Perhaps this desire reflects another important aspect: that the unique conditions of adolescents need to be the basis for advice if they are to be learned and incorporated. The advice needed might be different for each individual, as socioeconomic conditions, possible health conditions such as anxiety, depression, pain, and the family situation can vary and be of different importance. If there is an established relationship with the school nurse, health professional, or other reliable adults who provide advice or supportive education, the individual conditions can naturally be taken into account when establishing potential support. This reasoning reflects that it is important to evaluate what can be done in a group and what can best be done at an individual level.

This study indicates that sleep information or education in school was not perceived as engaging by the adolescents. However, they do wish to participate in sleep education and be interactive during the learning process, which is in line with pedagogical research showing that the best way to learn is via activities where students help each other gain knowledge (Race, 2020). Blunden and Rigney (2015), who reviewed sleep interventions performed at schools, also argue that if better success with behavioral changes is to be achieved, and not just an increase in knowledge, the pedagogy in the interventions needs to be reviewed. Including adolescents and their families in research can be a promising pathway to form interventions, since the involvement often challenges the usual beliefs and offers long-term quality and effective efforts (Carman et al., 2013).

The results also demonstrate that adolescents want to be guided into finding balance between the constant flow of information and silence. They are not accustomed to the silence that occurs when they go to bed, and this is perceived as threatening and difficult to handle. During this silence, it is common for existential concerns to take over. Hiller et al. (2013) stated that 87% of adolescents with sleep disorders reported catastrophic thinking in their pre-sleep period, such as concerns regarding the events of the day and planning for future events. Distracting themselves from the silence by using their mobile phones is their way of handling their thoughts, avoiding discomfort, and falling asleep eventually. Research shows that even if adolescents want to use their mobile phones, it is perceived as a barrier to sleep (Godsell & White, 2019; Hedin et al., 2020; Jakobsson et al., 2020; Quante et al., 2019). Adults could support adolescents in finding ways to deal with silence, provided the ways do not include making them reliant on their phones.

Adolescents also demonstrated a desire for support regarding activities and structure in order to achieve balance. There is evidence that support with time management strategies to reduce stress in school (Bauducco et al., 2020) promotes sleep. According to the adolescents in this study, school stress, which is a common reason for sleeping difficulties (Jakobsson et al., 2020), could be relieved through the support of a schedule for homework and tests and guidance regarding study techniques. In addition, as a support through structure in school, the adolescents in the present study described that exercise is positive for their sleep, but also for alertness at school, which is in line with previous research by Bartel et al. (2015). School management may consider the possibility of offering daily exercise in school. A benefit is that exercise organized through the school reaches all adolescents, regardless of socioeconomic conditions (Dai, 2019). With exercise or other practical lessons in the morning, according to the adolescents, performance in the remainder of the school day benefits: a mutually beneficial situation. To support adolescents’ sleep, a balance is needed during waking hours, which is something that can be arranged and facilitated by surrounding adults such as school nurses, teachers, and parents.

### 4.1 Implications for School Nursing

Since adolescents request individual support, a recommendation is that school nurses should be attentive to every individual adolescent’s sleep by genuinely listening to and asking about their own experiences. Sometimes the adolescents themselves are not aware that their sleep is insufficient and that they can ask for help, instead believing that their sleeping difficulties are simply consequences of adolescence (Jakobsson et al., 2022). If sleeping difficulties are experienced, it is helpful to let the individual fill in a sleep diary for one or two weeks; this provides a good basis for further conversation (Short et al., 2017).

One implication, which is in line with Khor et al. (2021) and Machado et al. (2020), is to involve the parents in sleep promotion. This involvement is for preventive purposes, among others, as parents’ support regarding routines, regularity, warmth, security, and home environment plays a key role in addressing sleep concerns. This can be done by utilizing the contact areas that already exist between school and parents, such as in parent meetings where they can be encouraged to be aware and proactive in their role as crucial supports for adolescents’ sleep.

A further implication is to promote increased knowledge in adolescents about the importance of sleep, the consequences of inadequate sleep, and, above all, tips and advice on how sleep can be improved. Advice that can be given to adolescents can include guidance about routines and regularity regarding bedtime, meals, daily physical activity, screen regulation before bedtime, relaxation before bedtime, spending time in the daylight, parental support, and a good family environment (Bartel et al., 2015; Khor et al., 2021; Machado et al., 2020). However, the advice must still be tailored to each unique adolescent.

Finally, school nurses should engage in issues related to the individuals’ balance between a variety of aspects, such as movement and rest, school and leisure, and the constant flow of information and silence. Here, school nurses can take actions to guide adolescents into balance of these aspects.

### 4.2. Strengths and Limitations

A strength of this study was the number of implemented focus groups, given the minimum requirement for two groups (Krueger & Casey, 2015). This enabled deep and varied data. Another strength was that the adolescents in the groups knew each other, making the conversational climate permissive and respectful. Another strength was the first author's experience as a former school nurse accustomed to leading adolescents’ conversation groups. The adolescents appreciated the ease of having conversations with someone who understood their context. In addition to the study's strengths, there were some limitations. The sample did not include adolescents who do not live with their parents, and although socioeconomic backgrounds varied, all participants had access to food, housing, and free schooling. The transferability of the results to adolescents living in more vulnerable contexts is thus limited. Since anxiety, depression, pain, and other ill health conditions can correlate with sleeping difficulties it can be a limitation that this is not included in the background descriptions. The sample was not randomized and the result is thus not generalizable. According to Krueger and Casey (2015), focus group interviews are not intended to be generalizable, the goal being to spend time listening to a smaller number of people in order to contribute to a deeper understanding of the phenomenon. Then, the reader decides the transferability, whether the results can be applied to other settings, contexts, or situations (Graneheim & Lundman, 2004) and, thus, be useful for supporting other adolescents’ sleep.

### 4.3 Conclusion

The support adolescents see as most helpful and necessary are parental involvement, knowledge of the “whys” and “hows” of sleep, and guidance on finding balance. According to the adolescents, the parents’ routines, security, and guidance play a key role in relation to their sleep. It is also the parents’ tips and advice that the adolescents listen to most. As a result, parents might need education to be able to guide their adolescents. Parents have an obvious role to play in relation to activities and finding a balance during the daytime. By involving parents more clearly, sleep education may be more successful. Further research should investigate how parental involvement ought to be composed; at the same time, it is important to take the adolescents’ increased independence into account. The adolescents’ suggestions of how to support sleep consistently include a desire to receive knowledge and support that is directed at them as individuals. For this purpose, adolescents need to be listened to; then the opportunity is given to influence and prevent the individual sleeping difficulties.
